# A novel liver zonation phenotype-associated molecular classification of hepatocellular carcinoma

**DOI:** 10.3389/fimmu.2023.1140201

**Published:** 2023-03-02

**Authors:** Tao Zhang, Jian Gu, Xinyi Wang, Yaoyao Lu, Kailin Cai, Huili Li, Yingli Nie, Xiangdong Chen, Jiliang Wang

**Affiliations:** ^1^ Department of Anesthesiology, Union Hospital, Tongji Medical College, Huazhong University of Science and Technology, Wuhan, China; ^2^ Department of Gastrointestinal Surgery, Union Hospital, Tongji Medical College, Huazhong University of Science and Technology, Wuhan, China; ^3^ Department of Dermatology, Wuhan Children’s Hospital (Wuhan Maternal and Child Healthcare Hospital), Tongji Medical College, Huazhong University of Science and Technology, Wuhan, China

**Keywords:** hepatocellular carcinoma, liver zonation, immunotherapy, single-cell sequencing, classification, prognosis, drug sensitivity, precision medicine

## Abstract

**Background:**

Liver zonation is a unique phenomenon in which the liver exhibits distinct functions among hepatocytes along the radial axis of the lobule. This phenomenon can cause the sectionalized initiation of several liver diseases, including hepatocellular carcinoma (HCC). However, few studies have explored the zonation features of HCC.

**Methods:**

Four single-cell RNA sequencing datasets were used to identify hepatocyte-specific zonation markers. Integrative analysis was then performed with a training RNA-seq cohort (616 HCC samples) and an external validating microarray cohort (285 HCC samples) from the International Cancer Genome Consortium, The Cancer Genome Atlas, Gene Expression Omnibus, and EMBL’s European Bioinformatics Institute for clustering using non-negative matrix factorization consensus clustering based on zonation genes. Afterward, we evaluated the prognostic value, clinical characteristics, transcriptome and mutation features, immune infiltration, and immunotherapy response of the HCC subclasses.

**Results:**

A total of 94 human hepatocyte-specific zonation markers (39 central markers and 55 portal markers) were identified for the first time. Subsequently, three subgroups of HCC, namely Cluster1, Cluster2, and Cluster3 were identified. Cluster1 exhibited a non-zonational-like signature with the worst prognosis. Cluster2 was intensively associated with a central-like signature and exhibited low immune infiltration and sensitivity toward immune blockade therapy. Cluster3 was intensively correlated with a portal-like signature with the best prognosis. Finally, we identified candidate therapeutic targets and agents for Cluster1 HCC samples.

**Conclusion:**

The current study established a novel HCC classification based on liver zonation signature. By classifying HCC into three clusters with non-zonational-like (Cluster1), central-like (Cluster2), and portal-like (Cluster3) features, this study provided new perspectives on the heterogeneity of HCC and shed new light on delivering precision medicine for HCC patients.

## 1 Introduction

Hepatocellular carcinoma (HCC) is one of the most common types of cancer worldwide (1). Despite the rapid progression of new diagnostic methods and therapeutic strategies for HCC, its prognosis is still unfavorable due to its high heterogeneity. Therefore, uncovering the molecular mechanisms underlying HCC diversity is essential for the development of targeted and effective therapies.

The liver is a central organ that maintains physiological homeostasis. Liver lobules are the functional units of the liver and are hexagonal in shape. Hepatocytes are organized in a cord-like arrangement along the liver lobule, extending from portal nodes (PN) to the central vein (CV) (2). According to the location of hepatocytes, the liver lobule can be divided into three zones: zone 1 (periportal area) is the region near the portal triad, zone 3 (pericentral zone) is near the CV, and the region between these zones is zone 2 (mid-lobular) (3). The liver shows functional partition along the lobule radial axis, a phenomenon known as “liver zonation”. Hepatocytes in different zonations perform various functions, allowing multiple of functions to proceed in parallel. Most of the liver’s metabolic functions take place in zone 1 hepatocytes, such as β-oxidation, gluconeogenesis, and lipid metabolism. On the contrary, zone 3 hepatocytes play central roles in glycolysis, xenobiotic biotransformation reactions, and glutamine synthesis (4).

Zonation is a dynamic process that plays essential roles in regulating liver disease phenotypes and progression. For example, since fatty acid synthesis and lipid accumulation occur predominantly in zone 3, whereas fatty acid oxidation occurs in zone 1. Patients with non-alcoholic fatty liver disease (NAFLD) often (approximately 37%) exibit perivenous dominant steatosis (5). In addition, patients with HCC exhibit impaired Wnt/β-catenin signaling (6), which stands out as a major regulator of liver zonation, regulating about a third of liver zonated genes (7).

In the past decades, genome-wide analyses have been devoted to deciphering the molecular mechanisms of HCC diversity (8–12). However, few studies have explored the molecular classification of HCC associated with liver zonation characteristics (11). In the present study, we constructed a novel molecular classification associated with liver zonation phenotype, and 3 subgroups of HCC, namely Cluster1, Cluster2, and Cluster3 were identified. We then evaluated the prognostic value, clinical characteristics, transcriptome and mutation features, immune infiltration, and immunotherapy response of the HCC subclasses. Finally, we identified candidate therapeutic targets and agents for Cluster1 HCC samples, which have the worst prognosis among the three clusters.

## 2 Materials and methods

### 2.1 Patients and samples

HCC patient cohorts with survival data were retrieved from several databases, including GEO (Gene Expression Omnibus), ICGC (International Cancer Genome Consortium), TCGA (The Cancer Genome Atlas), EMBL-EBI (EMBL’s European Bioinformatics Institute), and CPTAC (Clinical Proteomic Tumor Analysis Consortium). In total, five cohorts were enrolled, including TCGA-Liver Hepatocellular Carcinoma (TCGA-LIHC), ICGC-Liver Cancer-RIKEN-Japan (LIRI-JP), GSE14520, E-TABM-36, and CPTAC-HCC cohorts.

RNA sequencing data (counts) of 374 and 242 HCC human samples with available clinical information were obtained from the TCGA-LIHC cohort (https://xenabrowser.net/datapages/) and the LIRI-JP cohort (https://dcc.icgc.org/projects/LIRI-JP), respectively. The SVA R package was utilized to merge and remove the batch effects of the two RNA-seq datasets to create one metadata cohort using the Sangerbox online tool (13). Additional microarray data of 225 and 60 HCC samples from GSE14520 (https://www.ncbi.nlm.nih.gov/geo/) and E-TABM-36 (https://www.ebi.ac.uk/arrayexpress/files/E-TABM-36) based on the HG-U133A platform, were used for external validation. A metadata cohort was also created by merging the two microarray datasets, and batch effects were removed using the combat function in SVA R package. A boxplot before and after batch effect correction is shown in **Supplementary figure S1**.

We downloaded proteogenomics and clinical information from the CPTAC database (https://cptac-data-portal.georgetown.edu/) for 165 HCC tissues and 165 corresponding normal tissues.

Moreover, gene somatic mutation data (MAF files) of the TCGA-LIHC and LIRI-JP cohorts were obtained from TCGA and ICGC databases, respectively. Additionally, copy number data of GISTIC2 for the TCGA-LIHC cohort were accessed from the UCSC Xena (https://xenabrowser.net/datapages/).

Furthermore, the gene expression data and drug sensitivity data (AUC values) of hepatocellular carcinoma cell lines were downloaded from the DepMap database (https://depmap.org/portal/download/).

Additionally, 10x single-nuclei RNA-seq (snRNA-seq) data from 14 mouse and 9 human liver samples were retrieved from GSE192742 (https://www.ncbi.nlm.nih.gov/geo/).

### 2.2 Identification of hepatocyte-specified liver zonation markers

**Figure 1:**
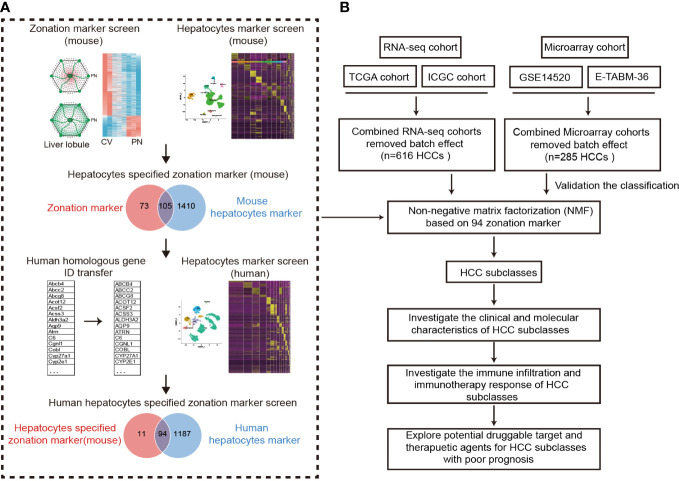
Design overview. **(A)** The procedure of the identification of hepatocyte-specified zonation markers. **(B)** The flowchart of the study. ICGC, International Cancer Genome Consortium; TCGA, The Cancer Genome Atlas; HCC, Hepatocellular carcinoma.

The procedure for identifying hepatocyte-specified liver zonation markers was shown in **Figure 1**. Halpern et al. and Moshe et al. combined single-molecule fluorescence *in situ* hybridization (smFISH) or fluorescence-activated cell sorting (FACS) of liver zonation surface markers with scRNA-seq to reveal the zonation patterns of hepatocyte gene expression (14, 15). Genes that were overexpressed in the PN area in both Halpern et al. and Moshe et al. cohorts were defined as portal markers. Meanwhile, genes that were overexpressed in the CV area in both Halpern et al.’s and Moshe et al.’s cohorts were defined as central markers.

To identify hepatocyte markers in both mice and humans, we utilized the Seurat R package (16) and applied standard downstream processing to mouse and human liver snRNA-seq data obtained from GSE192742 (17). Firstly, the LogNormalize method was applied for data normalization. Then, the “FindVariableFeatures” function (selection.method = “vst”, nfeatures = 3000) and the “FindIntegrationAnchors” function were using to select features and anchors for further integration. After integration, we eliminated low-quality cells with ≤ 500 genes or a mitochondrial gene ratio ≥ 5%. Finally, 25470 human liver cells and 25335 mouse liver cells that were deemed to be of good quality underwent further analysis. Following data integration and scaling, Seurat’s “RunPCA” function and “FindClusters” function were employed to perform principal component analysis and clustering. Dimensionality reduction was conducted utilizing the “RunUMAP” function, which utilized the top 30 calculated dimensions and a resolution of 0.5. Subsequently, the “FindAllMarkers” function was used to identify marker genes for each cluster (adjusted P < 0.05). Afterward, ToppGeneSuit (https://toppgene.cchmc.org/), CellMarker (http://xteam.xbio.top/CellMarker/), and PanglaoDB (https://panglaodb.se/search.html) databases were employed for the annotation of cell types. Finally, the “FindAllMarkers” function was employed to identify hepatocyte markers by comparing hepatocytes and other cell types (adjusted P < 0.05 and |logFC| > 0.25).

### 2.3 Identification of HCC subclasses

HCC samples of training and validating cohorts were classified using non-negative matrix factorization (NMF) clustering based on 94 liver zonation markers (18). And then, the prognosis of different subclasses in the training and validation cohorts was evaluated using the Kaplan-Meier log-rank test. Moreover, subclass mapping (SubMap) analysis (19) (Gene Pattern), a method for assessing the comparability of molecular classes between different patient cohorts based on their expression patterns, was then employed to verify whether the subclusters identified in the two cohorts were associated.

### 2.4 Molecular characteristics of HCC subclasses

The differentially expressed genes (DEGs) among the three clusters were identified using the “limma” package in R with cutoff criteria of |log2 fold change (FC)| > 1 and an adjusted P value < 0.05. Only genes that showed substantial differences in expression across all three comparisons were designated cluster-specific DEGs. Functional enrichment analysis of cluster-specific DEGs was conducted by the Metascape database (https://metascape.org/). P<0.05 was considered statistically significant.

Gene set variation analysis (GSVA) was then employed to estimate the score of the 50 hallmark gene sets that were achieved from the MsigDB database (http://www.gsea-msigdb.org/gsea/msigdb/index.jsp) (20). After that, we used the “ComplexHeatmap” R package to display distinct pathways among the three HCC clusters.

Moreover, Nearest template prediction (NTP) analysis (Gene Pattern modules) was used to predict the correlation between previously published HCC molecular classifications and our classification.

### 2.5 Estimation of immune infiltration and prediction of the immunotherapy response

To further explore the difference in immune cell infiltration among the clusters, the CIBERSORT algorithm (18) was used to estimate the fraction of 22 immune cell types in the HCC samples using Sangerbox online tool (13). In addition, single-sample GSEA (ssGSEA) was also used to estimate immune infiltration, which computed an enrichment score representing the degree to which genes in 28 immune cell gene sets were coordinately up or downregulated within a single sample (21).

To predict the response of immunotherapy in our subclasses, SubMap analysis (Gene Pattern) was employed to compare the similarity of gene expression profiles between our subclusters and a cohort of melanoma patients with programmed cell death protein-1 (PD1) inhibitor or cytotoxic T-lymphocyte-associated protein-4 (CTLA-4) inhibitor treatment (22).

### 2.6 Identification of potential drug targets and therapeutic agents for Cluster1 of HCC

The cluster1-specific DEGs with overexpression (log2FC > 1 and adjusted P < 0.05) in HCC tissues and low CERES scores (< -0.5) were defined as potential drug targets. The CERES scores were acquired from the dependency map (DepMap) portal (https://depmap.org/portal/). The CERES score is utilized to evaluate the dependency of the interest gene in a certain cancer cell lines, and a lower score suggests a higher likelihood that the gene is crucial for cell growth and survival of a given cancer cell line.

The Genomics of Drug Sensitivity in Cancer (GDSC) (23), and PRISM (24) databases contain information on drug sensitivity and gene expression profiles of cancer cells, which can be employed to establish a prediction model of drug response. The oncoPredict R package is used for predicting drug response through a ridge regression model to calculate sensitivity scores (low sensitivity score indicates high drug sensitivity) (25).

### 2.7 Statistical analysis

All computational and statistical analyses were performed using R programming (https://www.r-project.org/) and SPSS 22.0 (IBM Corp., Armonk, NY, USA). To compare two or three groups with normally distributed variables, the unpaired Student’s t-test and one-way ANOVA were utilized, respectively. Kruskal–Wallis tests were employed to compare three groups with non-normal distribution parameters. The chi-square test or Fisher’s exact tests were used for analyzing contingency table variables. Survival analysis was performed using Kaplan–Meier methods with the log-rank test. A two-tailed P value < 0.05 was statistically significant.

## 3 Results

### 3.1 Identification of hepatocyte-specified zonation markers

**Figure 2:**
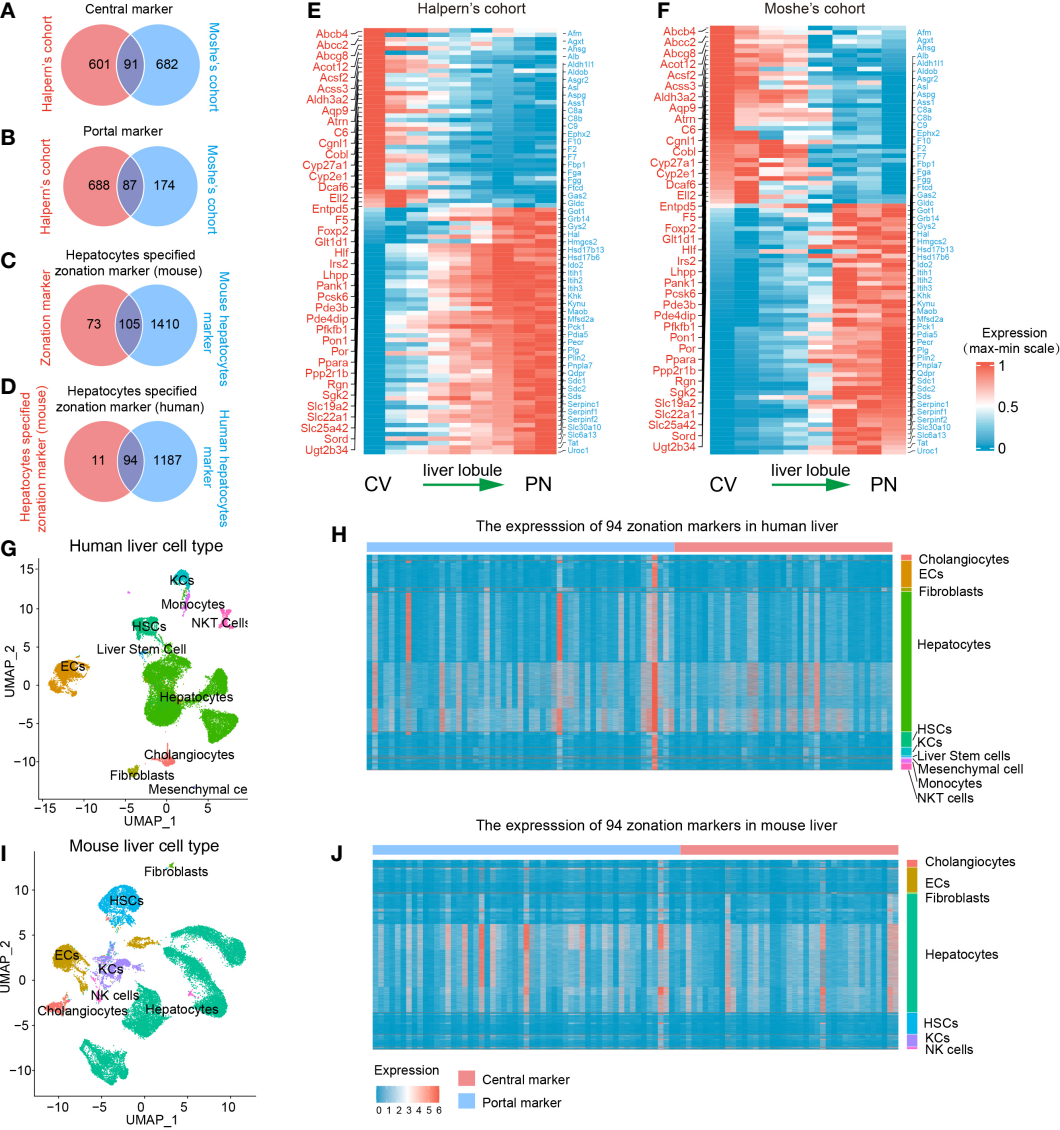
Identification of 94 human hepatocyte-specified zonation markers. Venn plots of central markers **(A)**, portal markers **(B)**, mouse hepatocyte-specified zonation markers **(C)**, and human hepatocyte-specified zonation markers **(D)**. Heatmap plots of the expression patterns of the 94 human hepatocyte-specified zonation markers in different areas of liver lobule in Halpern’s **(E)** and Moshe’s **(F)** cohorts. UMAP visualization of different cell types from human liver (25470 cells) by snRNA-seq **(G)**. Heatmap plot shows the expression of 94 hepatocyte-specified zonation markers in different cell types from the human liver **(H)**. UMAP visualization of different cell types from the mouse liver (25335 cells) by snRNA-seq **(I)**. Heatmap plot shows the expression of 94 hepatocyte-specified zonation markers in different cell types from mouse liver **(J)**. CV, Central venous; PN, Portal node; UMAP, Uniform Manifold Approximation and Projection; ECs, Endothelial cells; KCs, Kupffer cells; NK cells, Natural killer cells; HSCs, Hepatic stellate cells.

A flowchart was depicted to systematically describe the processes of identifying hepatocyte-specified zonation markers (**Figure 1A**). Firstly, we identified 1467 liver zonation-associated genes that were overexpressed in the CV or PN area in Halpern et al.’s cohort (**Table S1**). Moreover, 1034 liver zonation-associated genes were screened in Moshe et al.’s cohort (**Table S2**). Then, 91 central markers and 87 portal markers were identified, which were overexpressed in the CV and PN areas in both Halpern et al.’s and Moshe et al.’s cohorts, respectively (**Figures 2A, B**). Furthermore, we identified 1515 and 1281 mouse and human hepatocyte markers (**Tables S3**, **S4**). Afterward, 105 mouse hepatocyte-specified zonation markers were identified (**Figure 2C**). Finally, after transferring the 105 mouse zonation markers to human homologous genes, we screened 94 human hepatocyte-specified zonation markers, including 39 central markers and 55 portal markers (**Figure 2D**, **Table S5**). The expression pattern of the hepatocyte-specified zonation markers in the liver lobule was shown in **Figures 2E, F**. Moreover, the expression of the hepatocyte-specified zonation markers in different cell types of mouse and human liver was shown in **Figures 2G–J**.

Consistent with previous studies, functional enrichment analysis of these zonation markers indicated that central markers were mainly significantly enriched in processes of fatty acid metabolism, bile secretion, cholestasis, xenobiotic transport, glucose metabolism, and lipid localization. However, portal markers were mainly significantly enriched in processes of amino acid metabolism, complement and coagulation cascades, the urea cycle, regulation of proteolysis, gluconeogenesis, and the response to metal ion (**Table S6**).

### 3.2 NMF identifies three clusters in HCC

**Figure 3:**
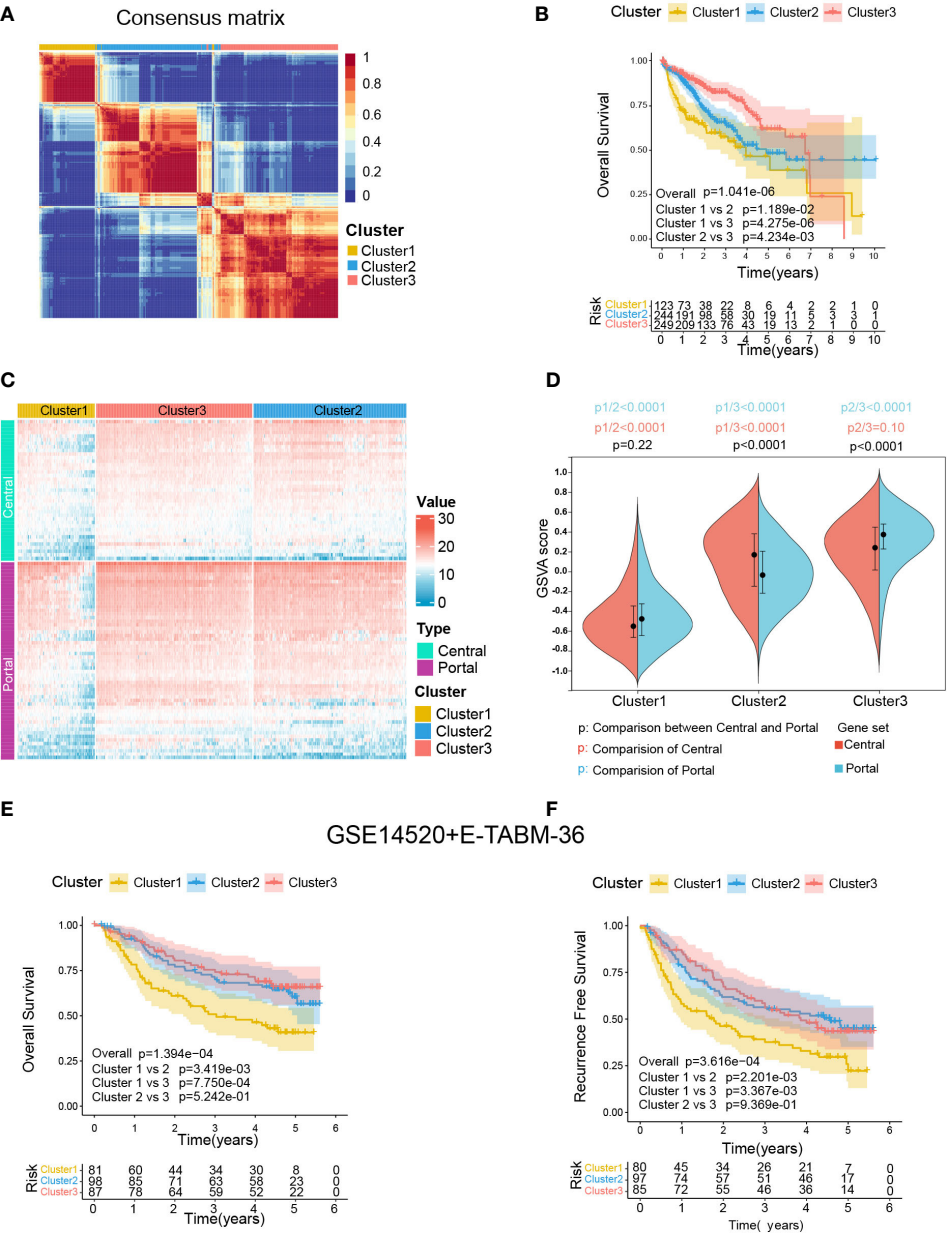
Identification of zonation marker-associated HCC subclasses using NMF clustering. **(A)** Heatmap plot shows the consensus matrix of NMF clustering results using the gene expression data of 94 zonation markers in the RNA-seq cohort (TCGA-LIHC+ICGC-LIRI-JP), colored by three HCC subclasses (Cluster1, Cluster2, and Cluster3). **(B)** Kaplan-Meier survival analysis of OS of the three clusters. **(C)** Heatmap plot shows the expression pattern of the zonation markers of the three clusters. **(D)** The difference of the GSVA scores of the portal and central signatures among the three clusters. Comparison between central and portal by Student’s t-test; Comparison among three clusters by ANOVA -Tukey test. **(E, F)** Kaplan-Meier survival analysis of OS and RFS of the three clusters from in microarray cohort (GSE14520+E-TABM-36). OS, Overall survival; RFS, Recurrence-free survival; GSVA, Gene set variation analysis.

The procedure of our bioinformatics analyses is shown in **Figure 1B**. The RNA-seq cohort comprising 616 HCC samples from TCGA-LIHC and ICGC-LIRI-JP were clustered based on the expression profile of 94 liver zonation markers using NMF consensus clustering. As shown in **Figure 3A**, three distinct clusters were identified in the RNA-seq cohort: Cluster 1 with 123 cases, Cluster 2 with 244 cases, and Cluster 3 with 249 cases. Moreover, a significant prognostic difference was observed among the three clusters in the RNA-seq cohort. Patients in Cluster1 had the worst prognosis, while patients in Cluster3 had the best prognosis (Overall log-rank test P = 1.041×10-6, Cluster1 vs 2 P = 1.189×10-2, Cluster1 vs 3 P = 4.275×10-6, Cluster2 vs 3 P = 4.234×10-3; **Figure 3B**). Subsequently, we explored the differences in the expression patterns of liver zonation markers among these clusters. As shown in **Figure 3C**, Cluster3 had the highest expression level of portal markers, while Cluster1 had the lowest expression level of both central and portal markers. Moreover, we performed GSVA analysis of gene sets based on central and portal markers to depict the zonation characteristics of each cluster. As shown in **Figure 3D**, consistent with gene expression level, Cluster3 had the highest GSVA score for portal markers, while Cluster1 had the lowest GSVA scores for both central and portal markers.

Subsequently, we conducted another independent analysis on a microarray cohort with 285 HCC samples from GSE14520 and E-TABM-36, the results of which also showed that there were three distinct clusters of HCC: Cluster 1 with 85 cases, Cluster 2 with 106 cases, and Cluster 3 with 94 cases (**Supplementary figure S2A**). Moreover, consistent with the RNA-seq cohort, a significant prognostic difference was observed among the three clusters in the microarray cohort, which patients in Cluster1 had the worst prognosis, and patients in Cluster2 and Cluster3 had better prognoses (Overall Survival: Overall log-rank test P = 1.394×10-4, Cluster1 vs 2 P = 1.394×10-4, Cluster1 vs 3 P = 7.750×10-4, Cluster2 vs 3 P = 5.242×10-1; **Figure 3E**; Recurrence Free Survival: Overall log-rank test P = 3.616×10-4, Cluster1 vs 2 P = 2.201×10-3, Cluster1 vs 3 P = 3.367×10-3, Cluster2 vs 3 P = 9.369×10-1; **Figure 3F**). Furthermore, as shown in **Supplementary figure S2C**, consistent with the RNA-seq cohort, Cluster3 in the microarray cohort had the highest expression level and GSVA score for portal markers, while Cluster1 had the lowest expression level and GSVA scores for both central and portal markers.

Finally, a SubMap analysis was performed to determine whether the clusters identified in the two above datasets were associated, and the result showed that Cluster1, Cluster2, and Cluster3 in the RNA-seq cohort were highly associated with corresponding clusters in the microarray cohort (P = 0.009), indicating there were three distinct molecular subclasses of HCC with different gene expression patterns (**Supplementary figure S2D**).

### 3.3 Correlation of the zonation-associated clusters with clinical characteristics and molecular subclasses of HCC published previously

**Figure 4:**
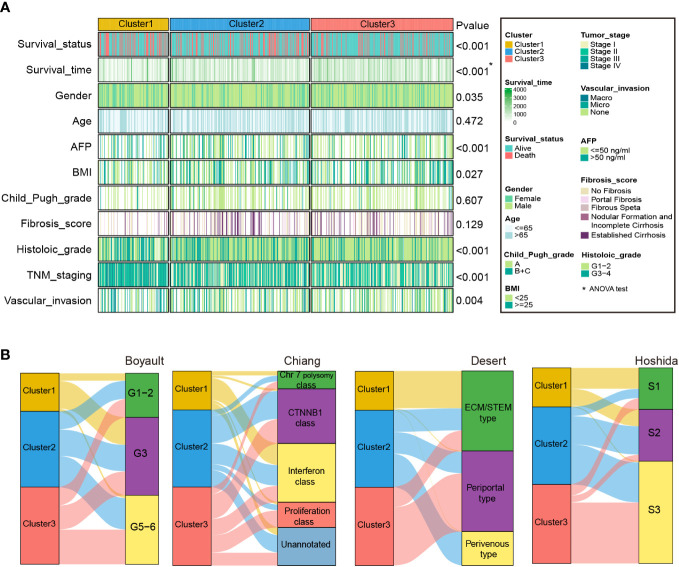
Clinical characteristics of zonation marker-associated HCC subclasses in the RNA-seq cohort. **(A)** Difference of clinical characteristics among the three clusters in the RNA-seq cohort by chi-square test (The comparison of survival time among the three clusters by ANOVA). **(B)** Correlation of the three clusters with HCC subclasses published previously in RNA-seq cohort by chi-square test.

We then explored the association between HCC-related clinicopathological variables and our classification based on the RNA-seq (**Figure 4A** and **Table S7**) and microarray cohorts (**Supplementary figure S3A** and **Table S8**). The chi-square and ANOVA tests indicated significant relationships between clinicopathological characteristics and HCC subtypes in the RNA-seq cohort. Favorable survival status (P < 0.001), longer survival time (P < 0.001), lower serum AFP level (P < 0.001), bigger BMI (P=0.027), lack of vascular invasion (P = 0.004), early TNM stage (P < 0.001), early histologic grade (P < 0.001), were associated with Cluster3, while worse survival status, shorter survival time (P < 0.001), smaller BMI, presence of vascular invasion, advanced histologic grade, advanced TNM stage, and high serum AFP level were associated with Cluster1. Similarly, in the microarray cohort, Cluster3 was correlated with favorable survival and recurrence status (P = 0.009, P = 0.045), longer survival and recurrence time (P < 0.001, P < 0.001), lower serum AFP level (P < 0.001), smaller tumor size (P = 0.01), and early TNM and BCLC staging (P = 0.005, P = 0.041). However, worse survival and recurrence status, shorter survival and recurrence time, smaller BMI, presence of vascular invasion, advanced histologic grade, advanced TNM stage, and high serum AFP level were associated with Cluster1.

In addition, we compared our categorization to previously identified HCC molecular subgroups, including Boyault’s classification (G1–G6) (8), Chiang’s classification (Chr 7 polysomy class, CTNNB1 class, Interferon class, Proliferation class, and Unannotated class) (9), Hoshida’s classification (S1, S2, and S3) (10), and Désert’s classification (ECM/STEM, Periportal, and Perivenous type) (11). In the RNA-seq cohort, the Cluster1 subclass was significantly associated with Boyault’s G3, Chiang’s Interferon class, Désert’s ECM/STEM type, and Hoshida’s S1. The Cluster2 subclass was linked to Désert’s Perivenous type and Hoshida’s S2. The Cluster3 subclass was associated with Chiang’s Proliferation class, Désert’s Periportal type, and Hoshida’s S3 (**Figure 4B** and **Table S7**). Similarly, in the microarray cohort, Cluster1 was linked to Chiang’s Interferon class, and Désert’s ECM/STEM type. Cluster2 was linked to Boyault’s G5-6, and Désert’s Perivenous type. Cluster3 was enriched in Boyault’s G5-6, Chiang’s Proliferation class, Désert’s Periportal, and Hoshida’s S3 (**Supplementary figure S3B** and **Table S8**).

### 3.4 Transcriptomes of the zonation-associated HCC clusters

Differential analyses were carried out to comprehend the distinctions in the molecular and biological processes among the three HCC subclasses. Significant differences in gene expression were defined as |log2FC| > 1 and the adjusted P-value < 0.05. Only genes that showed substantial differences in expression across all three comparisons were designated cluster-specific DEGs. Finally, 2682 specific DEGs (1868 upregulated and 814 downregulated) for Cluster1, 993 specific DEGs (118 upregulated and 875 downregulated) for Cluster2, and 405 specific DEGs (321 upregulated and 84 downregulated) for Cluster3 were identified (**Table S9**).

Next, functional enrichment analysis of the cluster-specific DEGs was performed utilizing the Metascape database, and remarkably enriched biological processes or pathways are shown in **Table S9**. The specific DEGs of the three clusters showed distinct enrichment of biological processes. Cluster1 was enriched in some differentiation and development-relevant processes. However, multiple metabolism-related biological processes and pathways were significantly enriched for Cluster3. For Cluster2, it was enriched in the processes associated with molecular transport and the WNT signaling pathway (**Table S10**).

**Figure 5:**
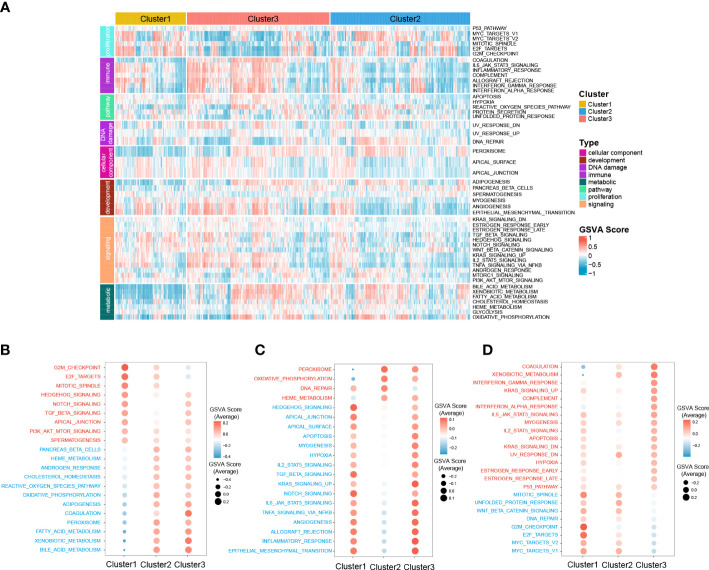
Difference of 50 hallmark gene sets among the three clusters. **(A)** Heatmap plot shows the GSVA score of 50 hallmark gene sets of the three clusters in RNA-seq cohort. The hyperactive and hypoactive processes or pathways in Cluster1 **(B)**, Cluster2 **(C)**, and Cluster3 **(D)**. Red represents hyperactive process or pathway and blue represents hypoactive process or pathway.

Moreover, to further investigate the molecular characteristics of the zonation marker-related subclasses, hallmark gene sets were chosen and quantified using the GSVA algorithm. Hallmark gene sets consist of eight process categories, including development, immune, signaling, cellular component, pathway, metabolic, DNA damage, and proliferation, which effectively condense the majority of the pertinent data from the initial sets and offer more precise and succinct inputs for gene set enrichment analysis (26). The result was presented in a heatmap (**Figure 5A**). Cluster1 exhibited high expression for proliferation relevant processes (e.g., G2M checkpoint, Mitotic spindle, and E2F targets), low expression for metabolic relevant processes (e.g., fatty acid metabolism, bile acid metabolism, and xenobiotic metabolism) (**Figure 5B**). Cluster2 exhibited low expression for immune (e.g., IL6-JAK-STAT3 signaling, Allograft rejection, and inflammatory response), development (e.g., Angiogenesis, Myogenesis, and Epithelial-mesenchymal transition), and signaling (e.g., IL2-STAT5 signaling, TNFA signaling *via* NFKB, TGFβ signaling, KRAS signaling up, Notch signaling, and Hedgehog signaling) relevant processes (**Figure 5C**). Cluster3 exhibited high expression for immune (e.g., Coagulation, Complement, Interferon-α response, and IL6-JAK-STAT3 signaling) and metabolic (e.g., Xenobiotic metabolism) relevant processes, low expression for proliferation relevant processes (e.g., G2M checkpoint, MYC targets, Mitotic spindle, and E2F targets) (**Figure 5D**).

### 3.5 The gene mutation profile of the zonation-associated HCC clusters

**Figure 6:**
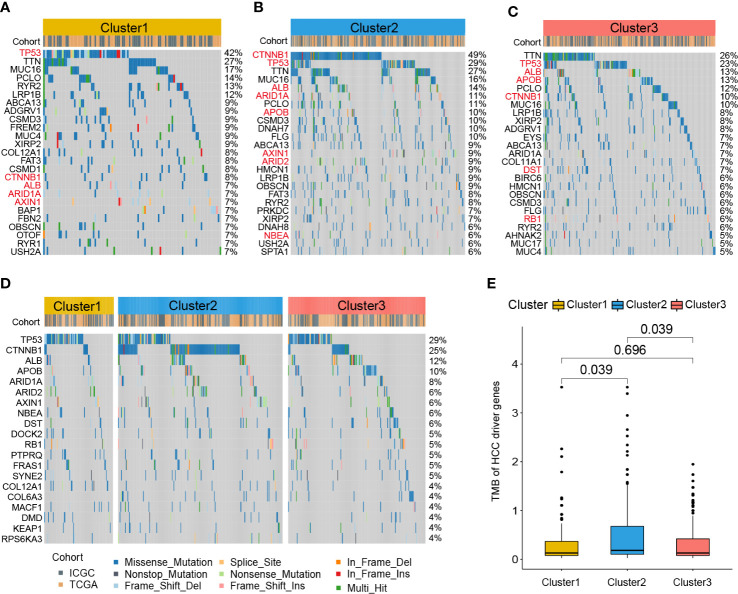
Gene mutation profile of zonation marker associated HCC clusters. Oncoplots of the top 25 somatic mutation genes in Cluster1 **(A)**, Cluster2 **(B)**, and Cluster3 **(C)**. Red represents HCC driver genes. Oncoplot of the top 20 somatic mutations of HCC driver genes **(D)**. Difference of TMB of HCC driver genes among the three clusters by Kruskal–Wallis test **(E)**. TMB, Tumor mutation burden.

To further investigate the different patterns of gene mutations among HCC clusters, somatic mutation data from the RNA-seq cohort was analyzed. The top 25 genes with the highest mutation rates in all three clusters were shown in **Figures 6A–C**. In Cluster1, TP53 exhibited the highest somatic mutation rate (42%), followed by TTN (27%), MUC16 (17%), PCLO (14%), RYR2 (13%), and LRP1B (12%). In Cluster2, CTNNB1 exhibited the highest somatic mutation rate (49%), followed by TP53 (29%), TTN (16%), MUC16 (14%), ALB (14%), ARID1A (11%), and PCLO (11%). For Cluster3, TTN exhibited the highest somatic mutation rate (26%), followed by TP53 (23%), ALB (13%), APOB (13%), PCLO (12%), CTNNB1 (10%), and MUC16 (10%).

A previous study identified 161 HCC driver genes that may play critical roles in the treatment of HCC (27). Therefore, we further explored the mutation profile of these driver genes. The top 20 driver genes with the highest mutation rates in all three clusters were shown in **Figure 6D**. TP53 exhibited the highest somatic mutation rate (29%), followed by CTNNB1 (25%), ALB (12%), APOB (10%), ARID1A (8%), ARID2 (8%), and AXIN1 (6%). Moreover, we compared the tumor mutation burden (TMB) of HCC driver genes among the three clusters. The results showed that Cluster2 exhibited a higher TMB level than the other two clusters (**Figure 6E**).

### 3.6 Correlation of the zonation-associated HCC clusters with immune infiltration and immunotherapy response

**Figure 7:**
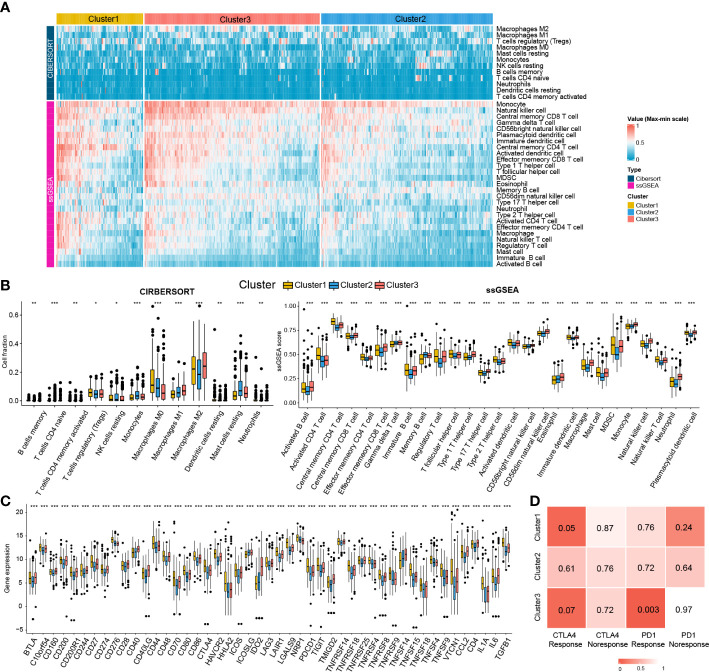
Immune cells infiltration landscape and immune checkpoint genes expression profile of three clusters. **(A)** Heatmap plot shows the abundance of immune cell infiltration in three clusters by CIBERSORT and ssGSEA. **(B)** Difference of the abundance of immune cells infiltration among different clusters by ANOVA. **(C)** Difference of the level of immune checkpoint genes among different clusters by ANOVA. *P<0.05; **P<0.01; ***P<0.001. **(D)** The SubMap matrix shows Cluster1 may be more sensitive to the CTLA-4 inhibitor (nominal P = 0.05) and Cluster3 may be more sensitive to the PD-1 inhibitor (nominal P = 0.003).

Given the significant differences in immune processes among clusters, immune infiltration was examined to characterize their immunological landscape. As shown in **Figure 7A**, the abundance of immune cell types was calculated using CIBERSORT and ssGSEA algorithms. As shown in **Figure 7B**, compared with the other two subclasses, Cluster2 presented the lowest abundance of 24 immune cell types, including B cells memory, macrophages M2, neutrophils, activated B cells, central memory CD4 T cell, central memory CD8 T cell, effector memory CD4 T cell, effector memory CD8 T cell, immature B cell, T follicular helper cell, regulatory T cell, Type 1/2/17 helper cell, activated dendritic cell, CD56dim natural killer cell, immature natural killer cell, macrophage, mast cell, MDSC, monocyte, natural killer T cell, natural killer cell, and Plasmacytoid dendritic cell. We further investigated the differences in the expression of 47 immune checkpoint genes among the three subclasses. Consistent with immune infiltration, Cluster2 exhibited the lowest expression for 44 immune checkpoint genes (except for ICOS, NRP1, and TNFSF14) compared to Cluster1 and Cluster3 (**Figure 7C**).

Different immune infiltration and immune checkpoint gene expression patterns among HCC clusters indicate that the immunotherapy response among the clusters may be different. Therefore, we compared the expression profiles of the three clusters (Cluster1, Cluster2, and Cluster3) with another published melanoma cohort containing 47 patients who received PD1 inhibitor or CTLA-4 inhibitor using SubMap analysis. As shown in **Figure 7D**, the expression profile of Cluster2 was correlated with the CTLA4 response group (P = 0.05), while Cluster3 was associated with the PD1 response group (P = 0.003), suggesting that patients within the Cluster1 group were more likely to respond to the anti-CTLA4 therapy and Cluster3 group was more likely to respond to anti-PD1 therapy.

### 3.7 Identification of potential drug targets and candidate therapeutic agents for Cluster1

**Figure 8:**
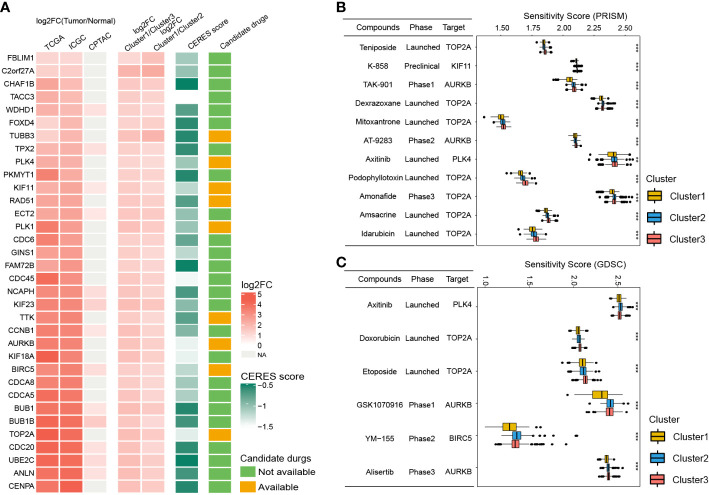
Identification of potential drug targets and candidate agents for Cluster1 HCC patients. **(A)** The 34 potential drug targets identified for Cluster1 HCC patients. **(B** and **C)** The sensitivity score, phase, and targets of candidate agents (eleven PRISM-derived compounds and seven GDSC-derived compounds) for Cluster1 HCC patients. **P<0.01; ***P<0.001.

Since patients in Cluster1 have the worst prognosis, we further identified the potential drug targets for Cluster1. The cluster1-specific DEGs with overexpression (log2FC > 1 and adjusted P < 0.05) in HCC tissues and low CERES score (< -0.5) were defined as potential drug targets. Eventually, a total of 34 potential drug targets were identified (**Figure 8A**, **Table S11**). Among them, drugs (compounds) that target AURKB, BIRC5, KIF11, PLK1, PLK4, RAD51, TOP2A, TTK, and TUBB3 were identified (**Figure 8A**, **Table S11**).

To further identify the potential therapeutic agents for Cluster1, the oncoPredict R package was used to calculate the sensitivity score of the drugs (compounds) for potential drug targets of Cluster1 based on the drug sensitivity and gene expression profiles of HCC cell lines from GDSC and PRISM databases. As shown in **Figures 8B, C**, eleven PRISM-derived compounds (including Teniposide, K-858, TAK-901, Dexrazoxane, Mitoxantrone, AT-9283, Axitinib, Podophyllotoxin, Amonafide, Amsacrine, and Idarubicin) and six GDSC-derived compounds (including Axitinib, Doxorubicin, Etoposide, GSK1070916, YM−155, and Alisertib) were found to be more sensitive in Cluster1 HCC patients.

## 4 Discussion

Liver zonation is a unique phenomenon that exhibits a distinct division of functions among hepatocytes along the lobule radial axis, optimizing overall liver function, and playing a central role in regulating liver disease phenotypes and progression, including HCC (28). Therefore, obtaining a better understanding of the characteristics of liver zonation in HCC may help us unveil the mechanisms of HCC heterogeneity and develop more effective therapeutics for HCC. With the breakthrough of RNA- sequencing technology, scRNA-seq coupled with spatial mapping has demonstrated previously unknown molecular patterns of hepatocytes, shedding new light on the functional features of hepatocytes across different zones of the liver lobule in both humans and mice, facilitating a better understanding of the form and regulation of zonation (29).

In the present study, we identified 94 hepatocyte-specified zonation markers (39 central markers and 55 portal markers) by combining four scRNA-seq cohorts for the first time. Based on the zonation markers, we identified three HCC subclasses. Cluster1 was barely involved in zonation-related signature, with a bad prognosis, high recurrence, high AFP level, and advanced TNM stage and histologic grade. Cluster2 was associated with a central signature, with a middle prognosis, AFP level, TNM stage, and histologic grade. Cluster3 exhibited a portal-associated signature, with favorable prognosis, low recurrence, low AFP level, and early TNM stage and histologic grade. In general, this study investigated the zonation characteristics of HCC, and identified three types of HCC with non-zonational-like (Cluster1), central-like (Cluster2), and portal-like (Cluster3) features, respectively.

Moreover, the transcriptome and mutation features, immune infiltration, and immunotherapy response of the subclasses were investigated. Cluster1 (non-zonational-like type) was mainly enriched in differentiation and development-relevant processes, with a high rate of TP53 mutation (42%), a high level of immune cell infiltration, a high expression level of immune checkpoint genes, and may be more sensitive to the CTLA4 inhibitor. Moreover, Cluster2 (central-like type) was mainly enriched in the processes associated with molecular transport and WNT signaling pathway, with a high rate of CTNNB1 mutation (49%), a low level of immune cell infiltration, and a low expression level of immune checkpoint genes. Cluster3 (portal-like type) was enriched in numerous metabolism-associated biological processes and pathways, with low rates of both TP53 (26%) and CTNNB1 (10%), a high level of immune cell infiltration, a high expression level of immune checkpoint genes, and may be more sensitive to the PD1 inhibitor.

Furthermore, we compared the correlation between our clusters and previously published HCC subclasses, which provided us with abundant characteristic information related to our novel zonation-associated clusters, leading to a deeper understanding of our clusters. The results from both RNA-seq and microarray cohorts demonstrated that Cluster1 was associated with the HCC type of Chiang’s Interferon class and Désert’s ECM/STEM type. Chiang’s Interferon class is featured by the overexpression of several interferon-stimulated genes, with a low mutation rate of CTNNB1 and low expression of IGF2 and CTNNB1 target genes (9). Désert’s ECM/STEM type is the collective name for the ECM type and STEM type. ECM type is characterized by ECM modeling, integrin signaling, and epithelial-mesenchymal transition. STEM type is typified by up-regulation of cell cycle progression and p53 mutation. ECM/STEM-type HCCs share high tumor cell proliferation and a bad prognosis (11). Cluster2 was linked to Désert’s Perivenous type. Désert’s Perivenous type is defined by a high level of perivenous hepatocyte signatures, such as fatty acid and bile salt metabolism, with a high rate of CTNNB1 mutations (11). Cluster3 was associated with Chiang’s Proliferation class, Désert’s Periportal type, and Hoshida’s S3. Chiang’s Proliferation class is characterized by high proliferation, chromosomal instability, activation of IGF and Akt/mTOR signaling, and reduced frequencies of CTNNB1 mutation (9). Désert’s Periportal type is featured by the enrichment of differentiated periportal hepatocyte signatures (gluconeogenesis, amino acid catabolism, hepatocyte nuclear factor 4A (HNF4A) induced genes) and TP53 mutation rates, with good prognosis, low recurrence, early-stage tumors by BCLC, TNM staging systems, and no macrovascular invasion (11). Hoshida’s S3 is notable for a well-differentiated hepatocyte signature with a good prognosis, and preserved TP53 function (10). In general, the clusters we constructed validated the findings of previously published HCC subclasses but also preserved their own characteristics.

HCC tumors are highly heterogeneous among individuals, and finding targeted therapeutic strategies for specific groups is of vital importance to maximize the therapeutic effect. In the present study, a total of 34 potential drug targets and 16 agents for Cluster1 HCC patients were identified. Among the potential drug targets, AURKB, BIRC5, KIF11, PLK1, PLK4, RAD51, TOP2A, TTK, and TUBB3 have targeted agents. Numerous studies have demonstrated the vital oncogenic role of AURKB (30), BIRC5 (31), KIF11 (32), PLK1 (33), PLK4 (34), RAD51 (35), TOP2A (36), TTK (37), and TUBB3 (38) in various cancers, including HCC. Therefore, multiple compounds have been designed for these therapeutic targets (**Table S11**). Among these compounds, Teniposide, K-858, TAK-901, Dexrazoxane, Mitoxantrone, AT-9283, Axitinib, Podophyllotoxin, Amonafide, Amsacrine, Idarubicin, Doxorubicin, Etoposide, GSK1070916, YM−155, and Alisertib were found to be more sensitive for Cluster1 HCC patients.

Teniposide, Dexrazoxane, Mitoxantrone, Podophyllotoxin, Amsacrine, Doxorubicin, and Etoposide are topoisomerase inhibitors that have been widely employed as chemotherapy in multiple cancer therapies. Amonafide is also a topoisomerase II inhibitor and DNA intercalator that has been found to have marked antineoplastic efficacy in preclinical models of cancer. Phase I and II studies revealed that Amonafide might be a promising drug for treating older patients, including those with multidrug-resistant, cytogenetically unfavorable secondary and treatment-associated acute myeloid leukemia (AML) (39).

TAK-901, AT-9283, GSK1070916, and Alisertib are Aurora kinase inhibitors. Human Aurora kinases, including Aurora kinase A (AURKA), B (AURKB), and C (AURKC), play critical roles in monitoring the mitotic checkpoint, bipolar mitotic spindle creation, and centrosome alignment, and also participate in regulating the separation of centrosome, bio-orientation of chromosomes, and cytokinesis. AURKB is a key regulator of mitosis and centrosome through polymerizing microfilaments and regulating chromatid segregation (40). Preclinical studies and Phase I/II/III trials have shown favorable pharmacokinetic properties and markedly antitumor effects of these agents (41–44).

K-858 is an inhibitor of the mitotic kinesin KIF11 (also known as Eg5). K-858 induces cell mitotic arrest with the formation of monopolar spindles by blocking centrosome separation, and activating the spindle checkpoint (45). K-858 has shown potent anti-proliferative and pro-apoptotic effects in multiple types of cancer cell lines (46, 47).

Axitinib is a potent and specific inhibitor of tyrosine kinase, specifically with high affinity for VEGFRs 1, 2, and 3, which was approved by the FDA for use in renal cell carcinoma in 2012 (48). In addition, Axitinib was also found to inhibit PLK4 with an IC_50_ value of 4.2 nM (49). Clinical trials have been performed to treat HCC patients with Axitinib. In a phase II trial, second-line treatment with Axitinib combined with the best supportive care led to a remarkably longer PFS and a higher clinical benefit rate than patients with only best supportive care in advanced HCC (50). Moreover, another multicenter phase II trial in advanced HCC revealed that second-line Axitinib treatment resulted in a 62.2% disease control rate and a 6.7% response rate for advanced HCC after failing the first-line sorafenib therapy (51).

YM-155 is an imidazolium-based compound that has selective activity against BIRC5 (also known as Survivin) (52). Several phase I/II clinical trials have demonstrated that YM-155 has a safe profile and anti-tumor capacity in multiple types of tumors (53, 54). Preclinical studies found that YM155 substantially suppressed the proliferation and induced cell cycle arrest and apoptosis of HCC cell lines. Moreover, in a mouse model using patient-derived HCC xenografts with overexpressed BIRC5 and p-BIRC5, YM155 exhibited stronger anti-proliferative efficacy than sorafenib (55).

## 5 Conclusion

In conclusion, the current study established a novel HCC classification based on liver zonation signature. By classifying HCC into three clusters with non-zonational-like (Cluster1), central-like (Cluster2), and portal-like (Cluster3) features, this study shed new lights on the heterogeneity of HCC. Cluster3 was intensively correlated with portal-like signature with a good prognosis. Cluster2 was intensively associated with a central-like signature and exhibited low immune infiltration and sensitivity towards immune blockade therapy. Cluster1 exhibited a non-zonational-like signature with the worst prognosis. Moreover, we identified potential drug targets and agents for Cluster1 HCC, which provided new insights into delivering precision medicine for HCC patients and shed new light on improving the therapeutic effect of anti-tumor drugs by selecting potentially sensitive patients.

## Data availability statement

The datasets presented in this study can be found in online repositories. The names of the repository/repositories and accession number(s) can be found within the article/**Supplementary materials**.

## Author contributions

TZ, and YN designed this work. JG, XW, and YL integrated and analyzed the data. HL and KC wrote this manuscript. TZ, YN, XC, and JW critical revision of the manuscript for important intellectual content, obtaining funding, and supervision. All authors contributed to the article and approved the submitted version.

## Supplementary material

Supplementary Figure 1 The Venn plot shows the common genes for TCGA-LIHC and ICGC-LIRI-JP cohorts **(A)**. The box plot shows the gene expression before and after batch effect correction of the RNA-seq cohort **(B)**. The Venn plot shows the common genes for GSE14520 and E-TABM-36 cohorts **(C)**. The box plot shows the gene expression before and after batch effect correction of the microarray cohort **(D)**

Supplementary Figure 2 Identification of zonation marker-associated HCC subclasses using NMF clustering. **(A)** Heatmap plot shows the consensus matrix of NMF clustering results using the gene expression data of 94 zonation markers in the microarray cohort (GSE14520+E-TABM-36), colored by three HCC subclasses (Cluster1, Cluster2, and Cluster3). **(B)** Heatmap plot shows the expression pattern of the zonation markers of the three clusters. **(C)** The difference of the GSVA scores of the portal and central signatures among the three clusters. Comparison between central and portal by Student’s t-test; Comparison among three clusters by ANOVA -Tukey test. **(D)** The SubMap matrix shows a significant correlation between HCC classification from RNA-seq and microarray cohorts. GSVA: Gene set variation analysis.

Supplementary Figure 3 Clinical characteristics of zonation marker associated HCC subclasses in the microarray cohort. **(A)** Difference of clinical characteristics among the three clusters in microarray cohort by chi-square test (The comparison of survival time among the three clusters by ANOVA). **(B)** Correlation of the three clusters with HCC subclasses published previously in microarray cohort by chi-square test.

## References

[1] Siegel RL Miller KD Jemal A. Cancer statistics, 2019. CA: Cancer J Clin (2019) 69(1):7–34. doi: 10.3322/caac.21551 30620402

[2] Manco R Itzkovitz S. Liver zonation. J Hepatol (2021) 74(2):466–8. doi: 10.1016/j.jhep.2020.09.003 33317845

[3] Rappaport AM. The microcirculatory acinar concept of normal and pathological hepatic structure. Beitrage zur Pathol (1976) 157(3):215–43. doi: 10.1016/s0005-8165(76)80083-2 1275864

[4] Paris J Henderson NC. Liver zonation, revisited. Hepatol (Baltimore Md) (2022) 76(4):1219–30. doi: 10.1002/hep.32408 PMC9790419 35175659

[5] Chalasani N Wilson L Kleiner DE Cummings OW Brunt EM Unalp A. Relationship of steatosis grade and zonal location to histological features of steatohepatitis in adult patients with non-alcoholic fatty liver disease. J Hepatol (2008) 48(5):829–34. doi: 10.1016/j.jhep.2008.01.016 PMC2346454 18321606

[6] Tian Y Mok MT Yang P Cheng AS. Epigenetic activation of wnt/β-catenin signaling in NAFLD-associated hepatocarcinogenesis. Cancers (2016) 8(8). doi: 10.3390/cancers8080076 PMC4999785 27556491

[7] Gebhardt R Matz-Soja M. Liver zonation: Novel aspects of its regulation and its impact on homeostasis. World J Gastroenterol (2014) 20(26):8491–504. doi: 10.3748/wjg.v20.i26.8491 PMC4093700 25024605

[8] Boyault S Rickman DS de Reyniès A Balabaud C Rebouissou S Jeannot E. Transcriptome classification of HCC is related to gene alterations and to new therapeutic targets. Hepatology (2007) 45(1):42–52. doi: 10.1002/hep.21467 17187432

[9] Chiang DY Villanueva A Hoshida Y Peix J Newell P Minguez B. Focal gains of VEGFA and molecular classification of hepatocellular carcinoma. Cancer Res (2008) 68(16):6779–88. doi: 10.1158/0008-5472.Can-08-0742 PMC2587454 18701503

[10] Hoshida Y Nijman SM Kobayashi M Chan JA Brunet JP Chiang DY. Integrative transcriptome analysis reveals common molecular subclasses of human hepatocellular carcinoma. Cancer Res (2009) 69(18):7385–92. doi: 10.1158/0008-5472.Can-09-1089 PMC3549578 19723656

[11] Désert R Rohart F Canal F Sicard M Desille M Renaud S. Human hepatocellular carcinomas with a periportal phenotype have the lowest potential for early recurrence after curative resection. Hepatology (2017) 66(5):1502–18. doi: 10.1002/hep.29254 28498607

[12] Yang C Huang X Liu Z Qin W Wang C. Metabolism-associated molecular classification of hepatocellular carcinoma. Mol Oncol (2020) 14(4):896–913. doi: 10.1002/1878-0261.12639 31955511 PMC7138397

[13] Shen W Song Z Zhong X Huang M Shen D Gao P. Sangerbox: A comprehensive, interaction-friendly clinical bioinformatics analysis platform. iMeta (2022) 1(3):e36. doi: 10.1002/imt2.36 PMC10989974 38868713

[14] Halpern KB Shenhav R Matcovitch-Natan O Toth B Lemze D Golan M. Single-cell spatial reconstruction reveals global division of labour in the mammalian liver. Nature (2017) 542(7641):352–6. doi: 10.1038/nature21065 PMC5321580 28166538

[15] Ben-Moshe S Shapira Y Moor AE Manco R Veg T Bahar Halpern K. Spatial sorting enables comprehensive characterization of liver zonation. Nat Metab (2019) 1(9):899–911. doi: 10.1038/s42255-019-0109-9 31535084 PMC6751089

[16] Stuart T Butler A Hoffman P Hafemeister C Papalexi E Mauck WM 3rd. Comprehensive integration of single-cell data. Cell (2019) 177(7):1888–902.e21. doi: 10.1016/j.cell.2019.05.031 31178118 PMC6687398

[17] Guilliams M Bonnardel J Haest B Vanderborght B Wagner C Remmerie A. Spatial proteogenomics reveals distinct and evolutionarily conserved hepatic macrophage niches. Cell (2022) 185(2):379–96.e38. doi: 10.1016/j.cell.2021.12.018 35021063 PMC8809252

[18] Gaujoux R Seoighe C. A flexible r package for nonnegative matrix factorization. BMC Bioinf (2010) 11:367. doi: 10.1186/1471-2105-11-367 PMC2912887 20598126

[19] Hoshida Y Brunet JP Tamayo P Golub TR Mesirov JP. Subclass mapping: identifying common subtypes in independent disease data sets. PloS One (2007) 2(11):e1195. doi: 10.1371/journal.pone.0001195 18030330 PMC2065909

[20] Hänzelmann S Castelo R Guinney J. GSVA: Gene set variation analysis for microarray and RNA-seq data. BMC Bioinf (2013) 14:7. doi: 10.1186/1471-2105-14-7 PMC3618321 23323831

[21] Charoentong P Finotello F Angelova M Mayer C Efremova M Rieder D. Pan-cancer immunogenomic analyses reveal genotype-immunophenotype relationships and predictors of response to checkpoint blockade. Cell Rep (2017) 18(1):248–62. doi: 10.1016/j.celrep.2016.12.019 28052254

[22] Roh W Chen PL Reuben A Spencer CN Prieto PA Miller JP. Integrated molecular analysis of tumor biopsies on sequential CTLA-4 and PD-1 blockade reveals markers of response and resistance. Sci Trans Med (2017) 9(379). doi: 10.1126/scitranslmed.aah3560 PMC5819607 28251903

[23] Iorio F Knijnenburg TA Vis DJ Bignell GR Menden MP Schubert M. A landscape of pharmacogenomic interactions in cancer. Cell (2016) 166(3):740–54. doi: 10.1016/j.cell.2016.06.017 PMC4967469 27397505

[24] Corsello SM Bittker JA Liu Z Gould J McCarren P Hirschman JE. The drug repurposing hub: a next-generation drug library and information resource. Nat Med (2017) 23(4):405–8. doi: 10.1038/nm.4306 PMC5568558 28388612

[25] Maeser D Gruener RF Huang RS. oncoPredict: An r package for predicting in vivo or cancer patient drug response and biomarkers from cell line screening data. Briefings Bioinf (2021) 22(6). doi: 10.1093/bib/bbab260 PMC8574972 34260682

[26] Liberzon A Birger C Thorvaldsdóttir H Ghandi M Mesirov JP Tamayo P. The molecular signatures database (MSigDB) hallmark gene set collection. Cell Syst (2015) 1(6):417–25. doi: 10.1016/j.cels.2015.12.004 PMC4707969 26771021

[27] Schulze K Imbeaud S Letouzé E Alexandrov LB Calderaro J Rebouissou S. Exome sequencing of hepatocellular carcinomas identifies new mutational signatures and potential therapeutic targets. Nat Genet (2015) 47(5):505–11. doi: 10.1038/ng.3252 PMC4587544 25822088

[28] Panday R Monckton CP Khetani SR. The role of liver zonation in physiology, regeneration, and disease. Semin Liver Dis (2022) 42(1):1–16. doi: 10.1055/s-0041-1742279 35120381

[29] Ramachandran P Matchett KP Dobie R Wilson-Kanamori JR Henderson NC. Single-cell technologies in hepatology: New insights into liver biology and disease pathogenesis. Nat Rev Gastroenterol Hepatol (2020) 17(8):457–72. doi: 10.1038/s41575-020-0304-x 32483353

[30] Borah NA Reddy MM. Aurora kinase b inhibition: A potential therapeutic strategy for cancer. Mol (Basel Switzerland) (2021) 26(7). doi: 10.3390/molecules26071981 PMC8037052 33915740

[31] Frazzi R. BIRC3 and BIRC5: multi-faceted inhibitors in cancer. Cell bioscience (2021) 11(1):8. doi: 10.1186/s13578-020-00521-0 33413657 PMC7792207

[32] Garcia-Saez I Skoufias DA. Eg5 targeting agents: From new anti-mitotic based inhibitor discovery to cancer therapy and resistance. Biochem Pharmacol (2021) 184:114364. doi: 10.1016/j.bcp.2020.114364 33310050

[33] Iliaki S Beyaert R Afonina IS. Polo-like kinase 1 (PLK1) signaling in cancer and beyond. Biochem Pharmacol (2021) 193:114747. doi: 10.1016/j.bcp.2021.114747 34454931

[34] Zhang X Wei C Liang H Han L. Polo-like kinase 4's critical role in cancer development and strategies for Plk4-targeted therapy. Front Oncol (2021) 11:587554. doi: 10.3389/fonc.2021.587554 33777739 PMC7994899

[35] Demeyer A Benhelli-Mokrani H Chénais B Weigel P Fleury F. Inhibiting homologous recombination by targeting RAD51 protein. Biochim Biophys Acta Rev Cancer (2021) 1876(2):188597. doi: 10.1016/j.bbcan.2021.188597 34332021

[36] Uusküla-Reimand L Wilson MD. Untangling the roles of TOP2A and TOP2B in transcription and cancer. Sci Adv (2022) 8(44):eadd4920. doi: 10.1126/sciadv.add4920 36322662 PMC9629710

[37] Serrano-Del Valle A Reina-Ortiz C Benedi A Anel A Naval J Marzo I. Future prospects for mitosis-targeted antitumor therapies. Biochem Pharmacol (2021) 190:114655. doi: 10.1016/j.bcp.2021.114655 34129859

[38] Duly AMP Kao FCL Teo WS Kavallaris M. βIII-tubulin gene regulation in health and disease. Front Cell Dev Biol (2022) 10:851542. doi: 10.3389/fcell.2022.851542 35573698 PMC9096907

[39] Freeman CL Swords R Giles FJ. Amonafide: a future in treatment of resistant and secondary acute myeloid leukemia? Expert Rev Hematol (2012) 5(1):17–26. doi: 10.1586/ehm.11.68 22272701

[40] Green MR Woolery JE Mahadevan D. Update on aurora kinase targeted therapeutics in oncology. Expert Opin Drug Discovery (2011) 6(3):291–307. doi: 10.1517/17460441.2011.555395 PMC3088914 21556291

[41] Farrell P Shi L Matuszkiewicz J Balakrishna D Hoshino T Zhang L. Biological characterization of TAK-901, an investigational, novel, multitargeted aurora b kinase inhibitor. Mol Cancer Ther (2013) 12(4):460–70. doi: 10.1158/1535-7163.Mct-12-0657 23358665

[42] Qi W Liu X Cooke LS Persky DO Miller TP Squires M. AT9283, a novel aurora kinase inhibitor, suppresses tumor growth in aggressive b-cell lymphomas. Int J Cancer (2012) 130(12):2997–3005. doi: 10.1002/ijc.26324 21796626

[43] Hardwicke MA Oleykowski CA Plant R Wang J Liao Q Moss K. GSK1070916, a potent aurora B/C kinase inhibitor with broad antitumor activity in tissue culture cells and human tumor xenograft models. Mol Cancer Ther (2009) 8(7):1808–17. doi: 10.1158/1535-7163.Mct-09-0041 19567821

[44] Durlacher CT Li ZL Chen XW He ZX Zhou SF. An update on the pharmacokinetics and pharmacodynamics of alisertib, a selective aurora kinase a inhibitor. Clin Exp Pharmacol Physiol (2016) 43(6):585–601. doi: 10.1111/1440-1681.12571 26999067

[45] Nakai R Iida S Takahashi T Tsujita T Okamoto S Takada C. K858, a novel inhibitor of mitotic kinesin Eg5 and antitumor agent, induces cell death in cancer cells. Cancer Res (2009) 69(9):3901–9. doi: 10.1158/0008-5472.Can-08-4373 19351824

[46] De Iuliis F Taglieri L Salerno G Giuffrida A Milana B Giantulli S. The kinesin Eg5 inhibitor K858 induces apoptosis but also survivin-related chemoresistance in breast cancer cells. Investigat New Drugs (2016) 34(4):399–406. doi: 10.1007/s10637-016-0345-8 26994617

[47] Marconi GD Carradori S Ricci A Guglielmi P Cataldi A Zara S. Kinesin Eg5 targeting inhibitors as a new strategy for gastric adenocarcinoma treatment. Mol (Basel Switzerland) (2019) 24(21). doi: 10.3390/molecules24213948 PMC6864856 31683688

[48] Umeyama Y Shibasaki Y Akaza H. Axitinib in metastatic renal cell carcinoma: beyond the second-line setting. Future Oncol (London England) (2017) 13(21):1839–52. doi: 10.2217/fon-2017-0104 28707479

[49] Liu ZH Lei Q Wei W Xiong L Shi YJ Yan GY. Synthesis and biological evaluation of (E)-4-(3-arylvinyl-1H-indazol-6-yl) pyrimidin-2-amine derivatives as PLK4 inhibitors for the treatment of breast cancer. Rsc Adv (2017) 7(44):27737–46. doi: 10.1039/c7ra02518a

[50] Kang YK Yau T Park JW Lim HY Lee TY Obi S. Randomized phase II study of axitinib versus placebo plus best supportive care in second-line treatment of advanced hepatocellular carcinoma. Ann Oncol Off J Eur Soc Med Oncol (2015) 26(12):2457–63. doi: 10.1093/annonc/mdv388 26386123

[51] Lin ZZ Chen BB Hung YP Huang PH Shen YC Shao YY. A multicenter phase II study of second-line axitinib for patients with advanced hepatocellular carcinoma failing first-line sorafenib monotherapy. Oncol (2020) 25(9):e1280–e5. doi: 10.1634/theoncologist.2020-0143 PMC7485356 32271494

[52] Majera D Mistrik M. Effect of sepatronium bromide (YM-155) on DNA double-strand breaks repair in cancer cells. Int J Mol Sci (2020) 21(24). doi: 10.3390/ijms21249431 PMC7763167 33322336

[53] Tolcher AW Mita A Lewis LD Garrett CR Till E Daud AI. Phase I and pharmacokinetic study of YM155, a small-molecule inhibitor of survivin. J Clin Oncol Off J Am Soc Clin Oncol (2008) 26(32):5198–203. doi: 10.1200/jco.2008.17.2064 PMC4879696 18824702

[54] Satoh T Okamoto I Miyazaki M Morinaga R Tsuya A Hasegawa Y. Phase I study of YM155, a novel survivin suppressant, in patients with advanced solid tumors. Clin Cancer Res an Off J Am Assoc Cancer Res (2009) 15(11):3872–80. doi: 10.1158/1078-0432.Ccr-08-1946 19470738

[55] Xia H Chen J Shi M Deivasigamani A Ooi LL Hui KM. The over-expression of survivin enhances the chemotherapeutic efficacy of YM155 in human hepatocellular carcinoma. Oncotarget (2015) 6(8):5990–6000. doi: 10.18632/oncotarget.3337 25714025 PMC4467416

