# Supplementary figures and images for: A novel liver zonation phenotype-associated molecular classification of hepatocellular carcinoma
Source: Front Immunol. 2023 Mar 2;14:1140201. doi: 10.3389/fimmu.2023.1140201 (PMC10017747; doi:10.3389/fimmu.2023.1140201)

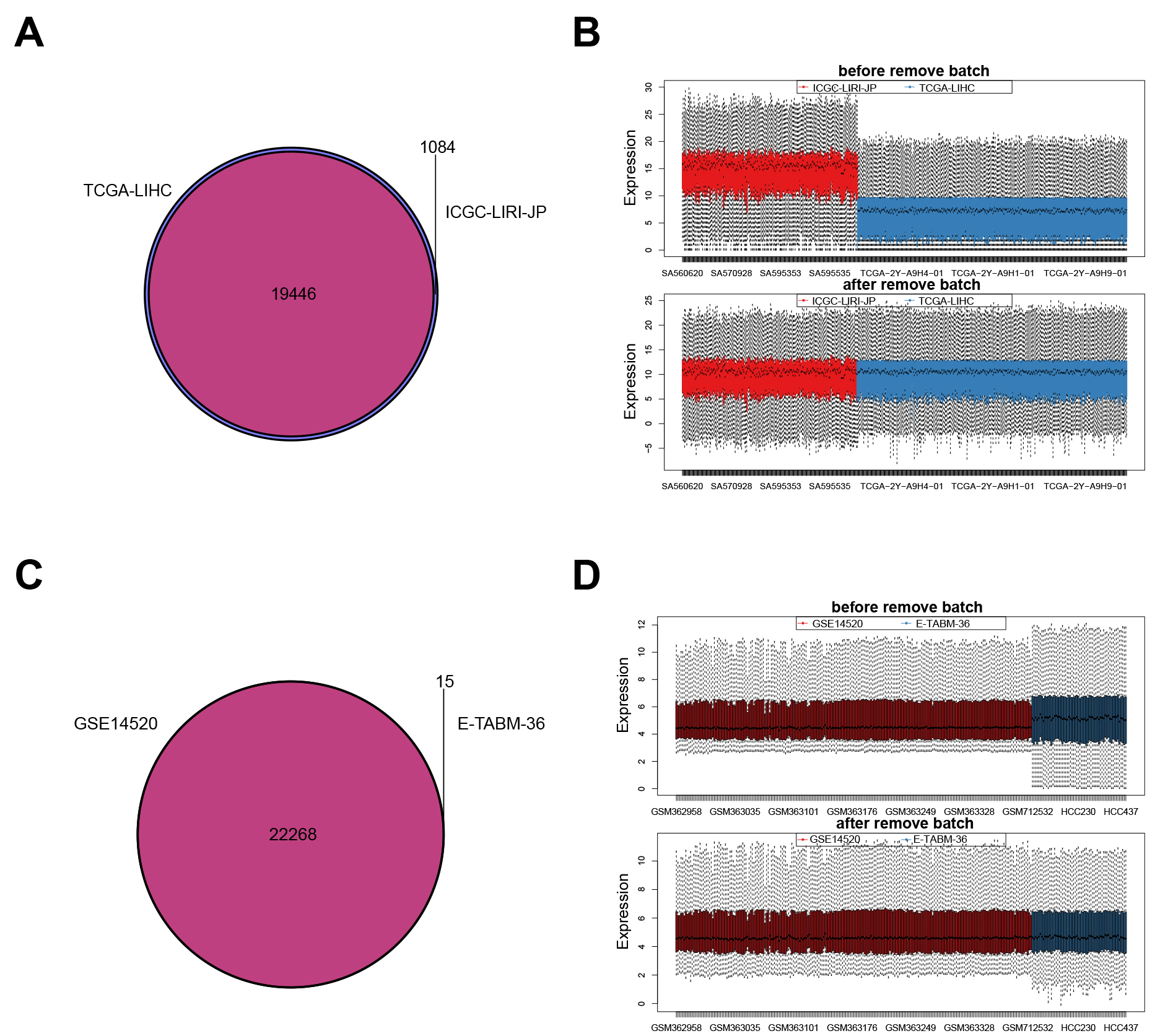

Supplement: Supplementary Figure 1 — The Venn plot shows the common genes for TCGA-LIHC and ICGC-LIRI-JP cohorts (A). The box plot shows the gene expression before and after batch effect correction of the RNA-seq cohort (B). The Venn plot shows the common genes for GSE14520 and E-TABM-36 cohorts (C). The box plot shows the gene expression before and after batch effect correction of the microarray cohort (D) [file Image_1.jpeg]

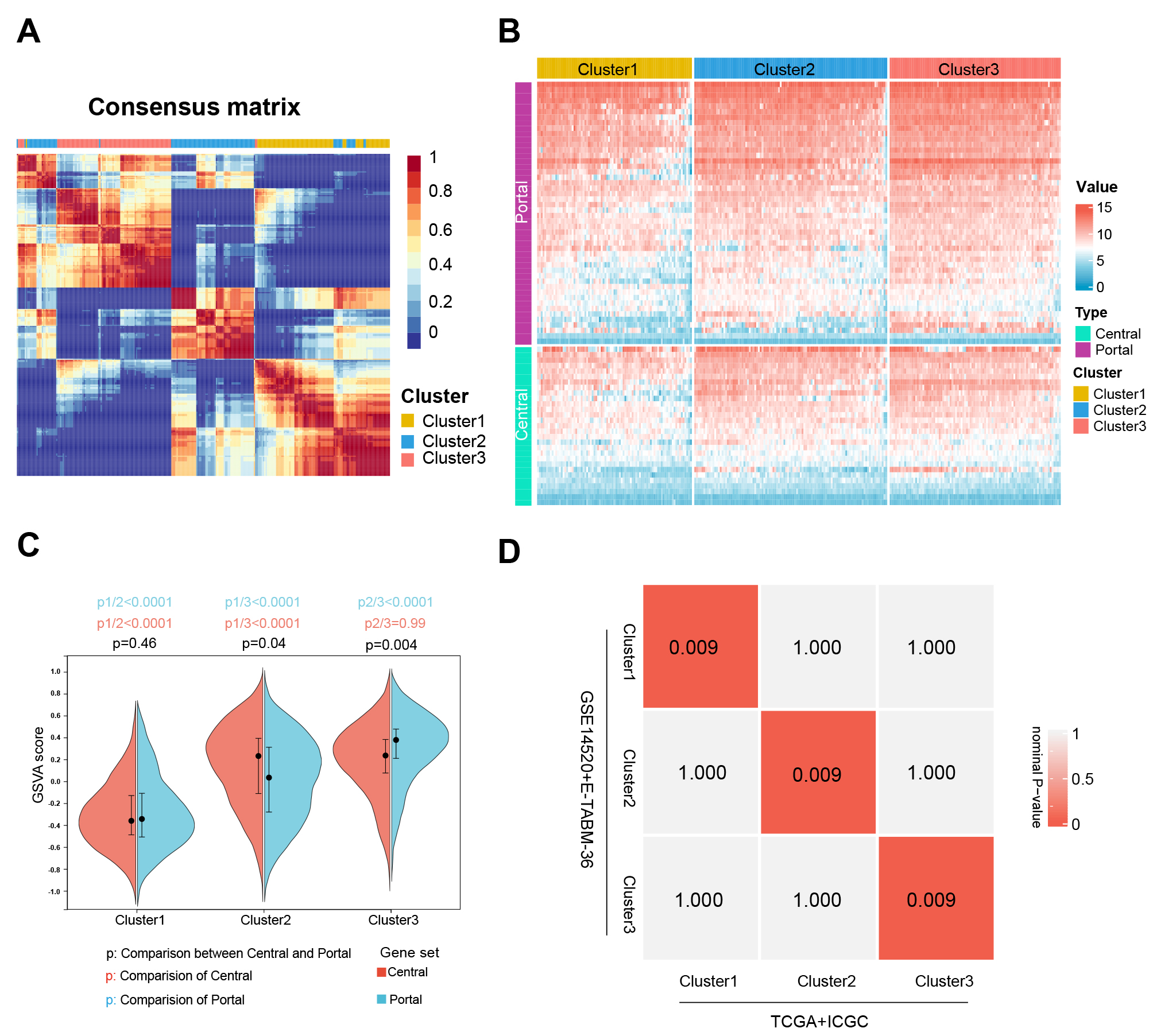

Supplement: Supplementary Figure 2 — Identification of zonation marker-associated HCC subclasses using NMF clustering. (A) Heatmap plot shows the consensus matrix of NMF clustering results using the gene expression data of 94 zonation markers in the microarray cohort (GSE14520+E-TABM-36), colored by three HCC subclasses (Cluster1, Cluster2, and Cluster3). (B) Heatmap plot shows the expression pattern of the zonation markers of the three clusters. (C) The difference of the GSVA scores of the portal and central signatures among the three clusters. Comparison between central and portal by Student’s t-test; Comparison among three clusters by ANOVA -Tukey test. (D) The SubMap matrix shows a significant correlation between HCC classification from RNA-seq and microarray cohorts. GSVA: Gene set variation analysis. [file Image_2.jpeg]

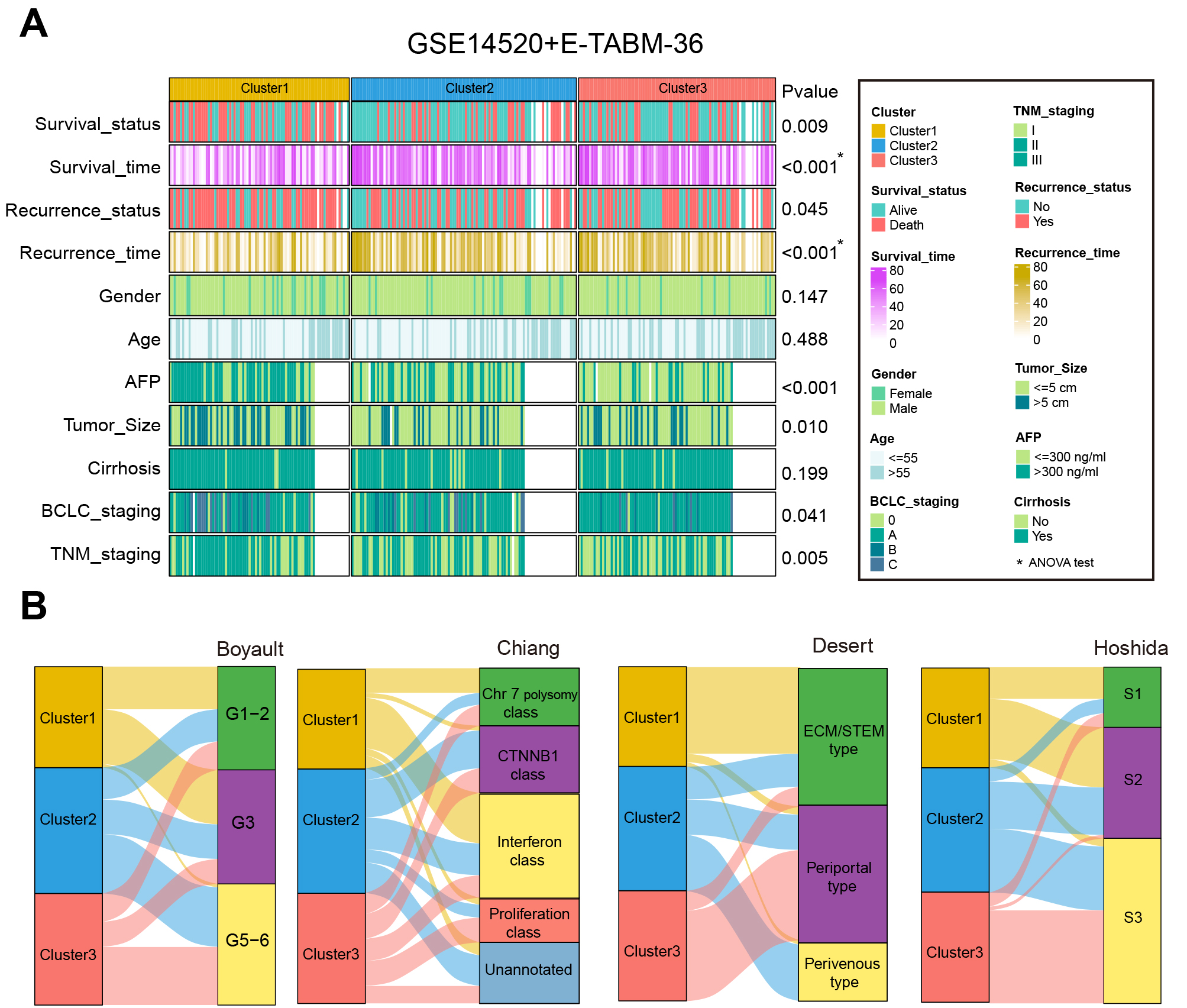

Supplement: Supplementary Figure 3 — Clinical characteristics of zonation marker associated HCC subclasses in the microarray cohort. (A) Difference of clinical characteristics among the three clusters in microarray cohort by chi-square test (The comparison of survival time among the three clusters by ANOVA). (B) Correlation of the three clusters with HCC subclasses published previously in microarray cohort by chi-square test. [file Image_3.jpeg]
